# A case study in pathway knowledgebase verification

**DOI:** 10.1186/1471-2105-7-196

**Published:** 2006-04-08

**Authors:** Stephen A Racunas, Nigam H Shah, Nina V Fedoroff

**Affiliations:** 1 Computational Learning Laboratory, Stanford University, CA, USA; 2 Stanford Medical Informatics, Stanford University, CA, USA; 3 Huck institutes of the Life Sciences, University Park, PA, USA

## Abstract

**Background:**

Biological databases and pathway knowledgebases are proliferating rapidly. We are developing software tools for computer-aided hypothesis design and evaluation, and we would like our tools to take advantage of the information stored in these repositories. But before we can reliably use a pathway knowledgebase as a data source, we need to proofread it to ensure that it can fully support computer-aided information integration and inference.

**Results:**

We design a series of logical tests to detect potential problems we might encounter using a particular knowledgebase, the Reactome database, with a particular computer-aided hypothesis evaluation tool, HyBrow. We develop an explicit formal language from the language implicit in the Reactome data format and specify a logic to evaluate models expressed using this language. We use the formalism of finite model theory in this work. We then use this logic to formulate tests for desirable properties (such as completeness, consistency, and well-formedness) for pathways stored in Reactome.

We apply these tests to the publicly available Reactome releases (releases 10 through 14) and compare the results, which highlight Reactome's steady improvement in terms of decreasing inconsistencies. We also investigate and discuss Reactome's potential for supporting computer-aided inference tools.

**Conclusion:**

The case study described in this work demonstrates that it is possible to use our model theory based approach to identify problems one might encounter using a knowledgebase to support hypothesis evaluation tools. The methodology we use is general and is in no way restricted to the specific knowledgebase employed in this case study. Future application of this methodology will enable us to compare pathway resources with respect to the generic properties such resources will need to possess if they are to support automated reasoning.

## Background

There has been a tremendous increase in the volume and variety of data about biological systems and many biological databases have been developed for storing and querying the rapidly accumulating data[1]. There are increasing efforts to create databases that allow representation of biological processes at higher levels of abstraction.

In particular, knowledgebases which represent biological pathways in an abstracted, structured manner are being developed on a large scale [2-4]. Such pathway knowledgebases will enable qualitative modelling and reasoning about large biological systems using computational tools for thought[4,5].

Knowledgebase developers realize the importance of providing reliable, complete, and consistent knowledgebases. For example, the pathway hole filler algorithm developed by Green et al attempts to find missing enzymes in automatically generated metabolic pathway databases[6] and fills them using information about sequence homology and annotation[7]. Methods have also been developed to compare the differential content in metabolic databases, using Petri nets to capture pathway representations from different databases[8]. This paper complements such efforts and presents methods to evaluate the ability of a pathway knowledgebase to support computer-aided information integration and inference tools[9]. Another key difference is that this work also provides a general methodology for proofreading knowledgebases, one based upon the formalism of model checking.

### HyBrow, Reactome and proofreading knowledgebases

Locating, retrieving and integrating biological data have become increasingly burdensome tasks, leading to a growing need for tools that offer an organizing framework to facilitate the interpretation of biological data[10]. We are developing such a tool, which we have called the Hypothesis Space Browser (HyBrow). In previous work, we described a HyBrow prototype implementation that uses a small knowledgebase containing information about the yeast galactose regulatory gene network[9]. We would like to use HyBrow in conjunction with the larger, more sophisticated knowledge repositories (such as Reactome) that are currently being developed[2].

The Reactome project is a collaborative effort involving the Cold Spring Harbor Laboratory, the European Bioinformatics Institute, and the Gene Ontology Consortium. Reactome is a knowledgebase containing representations of the core pathways and reactions in human biology, authored by expert biological researchers and maintained by the Reactome editorial staff. The basic unit of Reactome is the reaction, which Reactome defines as any biological event that converts inputs to outputs[2] where the inputs and outputs of a reaction are biological entities such as small molecules, proteins, lipids, nucleic acids, or complexes of these. Reactions include the chemical conversion of one set of entities to another as well as the formation and dissociation of complexes and the transport of entities from one compartment to another. During the construction of the knowledgebase, biologist domain experts assigned the individual reactions stored in Reactome to pathways.

The existing HyBrow prototype evaluates hypotheses on the event scale by checking the individual events and the logical combinations of events in each hypothesis for agreement with stored information. In the case of Reactome, we cannot check individual reactions, as they are provided "as is" by domain experts and the corresponding primary data are not stored. However, if we assume that the individual reactions are valid, we can check higher order properties for pathways. This exercise has dual benefits: we not only obtain a quality check for an important knowledgebase, but also develop methods that we can use in the future for principled evaluation of higher-order hypotheses. We propose logical proofreading as a pre-processing step to precede the use of a pathway database for other automated inference procedures. This would bring several advantages. For example, if a knowledgebase contains inconsistencies or gaps, automated pathway tracing can encounter ambiguities or dead-ends, causing problems for programs attempting to investigate possibilities for metabolic engineering. Logical proofreading facilitates human reasoning as well as automated reasoning. Human curation often encounters complications if multiple curators are involved. Logical proofreading can help by, for instance, identifying instances where two differing opinions exist, or where one curator assumed that a particular relationship was recorded elsewhere and thereby chose to record neither this assumption nor the original relationship.

### Investigation summary

Biologists will increasingly need tools for thought[5], and so we must develop tests to determine whether or not a given knowledgebase can support tools of this kind. It is possible to use a portion of the HyBrow logical framework[11] to check an existing knowledgebase for internal consistency and other desirable properties that would ensure its compatibility with machine-aided inference tools. We discussed this possible application with the Reactome development team to secure their support for a test case involving HyBrow and Reactome. We then developed a method for proofreading Reactome that is based upon composition and evaluation of second-order hypotheses in the HyBrow framework. We used this method to identify issues in past releases of Reactome and to design scripts that will be used by the Reactome team to troubleshoot new releases. We believe that there is a need for logical verification of structured molecular biology data resources, and this paper represents a first step in that direction. In future work, we will move beyond this case study to define a generic set of desirable knowledgebase properties and then evaluate multiple knowledgebases with respect to their expressive power and conformance with these properties.

## Results

Using the event-level relationships (precedence, supply and enablement) described in the methods section, we defined a set of testable desirable properties that we believe should hold for pathway-models stored in Reactome. These properties were formulated to identify errors that can cause problems for computer-aided information integration and inference tools such as HyBrow.

### Testable properties for pathways

1. We say that a sequence of events in a pathway representation is *well-formed *if the direct precedence relation holds whenever the direct supply relation holds over the events in the sequence.

2. We say a pathway representation is *complete *if every event in the pathway is either supplied by a preceding event or supplied and enabled by the axioms.

3. We say that an acyclic pathway representation is *inconsistent *if an event comes before another event in the pathway order, but the opposite is true in the precedence order.

4. We say that a pathway representation is *verbose *if there exist events a and c such that (a ≺ c) in the precedence order, and there exist "extra" events e_i_, i ≥ 1, such that (a ≺ e_1_), (e_1 _≺ e_2_) ... (e_i _≺ c) in the pathway ordering.

5. We say that a representation pathway is *terse *if there exist events a and c such that (a ≺ c) in the pathway ordering, and there exist "extra" events e_i_, i ≥ 1, such that (a ≺ e_1_), (e_1 _≺ e_2_) ... (e_i _≺ c) in the precedence order.

6. We say that a pathway representation is *gapped *if there exist events in the pathway for which supply is violated, and there exist preceding events in the database which are capable of supplying those pathway events.

7. We say that a sequence of events in a pathway representation is *unfounded *if there exist cycles in the precedence order that are not also defined to be cycles by the pathway annotation.

### Desirable properties

In order for Reactome to become a reliable resource of core pathways and reactions for automated inference about human biology, it is greatly desirable that its representations satisfy completeness, consistency and gap-free properties:

• Each pathway in the Reactome database should be complete. If a pathway is incomplete, a reasoner is unable to "backtrace" reactants to find their requisite precursors.

• None of the pathways in Reactome should be inconsistent. If a pathway is inconsistent, it is possible for a reasoner to arrive at two conflicting event orders. In the absence of further information, the reasoner would not be able to tell which of two events could possibly be a cause for the other.

• None of the pathways in Reactome should be gapped pathways. If a pathway contains gaps, a reasoner cannot verify the presence of all of the possible output entities that should be available at the end of the pathway.

Of less crucial importance are directness, well-formedness and well-foundedness properties:

• The directness criterion requires that as many pathways as possible should be adjusted so that they are neither verbose nor terse. This will enable reasoners that work with both the curated (pathway) and stored (precedence) data to get compatible information from both sources. This compatibility is essential if we want to "borrow" a pathway representation, based on precedence, from one organism and compare it to a curated pathway representation from another organism.

• The well-formedness criterion states that every sequence of events in Reactome should be a well-formed sequence. If not, this is a cue that precedence data are missing.

• The well-foundedness criterion mandates that there should not be any cyclical sequences in the database that are not also annotated to be cyclical pathways and that for no events should the precedence relation be reflexive. An unlabeled cycle in precedence or a reflexive precedence relation (a self-loop) can lead to non-terminating traces or require cutting the cycle at an arbitrary point.

### Proofreading Reactome

We developed scripts (available for download[12]) to test the concrete human pathways stored in Reactome for these properties. We focused on concrete pathways because the properties we have defined and the results of our analyses are easily interpreted for concrete pathways. This section summarizes the results of tests performed on release 10 of Reactome, the earliest publicly available release.

### Supply and enabling

Direct supply is often violated, due to missing events, the incomplete state of current experimental knowledge, and lapses in annotation.

For example, the pathway *CDK-mediated phosphorylation and removal of Cdc6 *(id 69017) has four events identified by ids: 69005, 69006, 69015, and 69016, of which the event, *Cytoplasmic phosphorylated Cdc6 is ubiquitinated by the anaphase-promoting complex *(id 69015), is unsupplied. This event requires Ubiquitin (id 113595) as an input, which is not supplied by any other reaction. Ubiquitin is also *not *identified as one of the concrete simple entities that are assumed to be present. To remedy this, Ubiquitin would have to be accounted for as a product of another reaction, or else it must be declared to be a concrete simple entity.

The concept of enabling is not yet tracked by Reactome, even though there are events that create physical entities which act as catalysts in other pathways. No events specifically modify or create catalysts for other events in a pathway.

### Data format integrity

Overall, Reactome adheres to its data format specification. However, generic entities are allowed to interact with concrete physical entities in generic pathways. This leads to peculiarities such as a generic event preceding a concrete event *e*, which precedes events that are *instances of *the generic event preceding *e*.

For example, the concrete reaction identified by id 111746 has the generic reaction 111742 as a preceding reaction. However, the concrete instances of this generic reaction (ids 111741, 111753, 111754, 111755) have reaction 111746 as their preceding event and thus we have 111742 ≺ 111746 ≺ (111741, 111753, 111754, 111755).

These reactions describe how the enzyme complex ribonucleotide reductase catalyzes the reduction of each of four nucleoside diphosphates, ADP, CDP, GDP, and UDP to the corresponding deoxynucleoside diphosphates, dADP, dCDP, dGDP, and dUDP. These reactions are represented by ids 111741, 111753, 111754, 111755. In the course of doing so, the enzyme complex itself is oxidized, and must be regenerated by reduction, before it can catalyze another round of ribonucleotide reduction. There are two ways of carrying out this reduction, one of which is in a reaction involving glutathione (reaction id 111746).

The reactome curators created a generic reaction, named *Reduction of cytosolic ribonucleoside 5'-diphosphates to deoxyribonucleoside 5'-diphosphates (glutaredoxin) *(id 111742) with four concrete instances (ids 111741, 111753, 111754, 111755) and linked the concrete regeneration event (id 111746) to this generic reduction reaction. This leads to the case where 111742 ≺ 111746 ≺ (111741, 111753, 111754, 111755). We pointed this out to the Reactome team and they agreed that this was indeed a problem.

This situation should ideally have been annotated as four cycles involving the four concrete reduction events (ids 111741, 111753, 111754, 111755) and the concrete regeneration event for each case.

To fix the problem, the curators decided to delete the "preceding event" link between the generic reduction reaction (id 111472) and the concrete regeneration reaction (id 111476), and replace it (as of version 14) with links directly from the concrete reduction to concrete regeneration events (ids 111741, 111753, 111754, 111755).

### Completeness

Since we found that Reactome does not consider enabling, we decided to include an assumption of universal enablement. (If we had not done so, the completeness criterion would be violated everywhere, obviating the diagnostic power of this test.) Reactome also does not track the creation and use of concrete simple entities, so we had to assume the availability of such entities to avoid systemic judgments of incompleteness. We also assumed the presence of the input reactants to the first step in each pathway.

After incorporating these assumptions, we were able to locate several pathways that were not complete. One such pathway is the inosine formation pathway.

The *inosine formation *pathway (id 74236) has two reactions: *Adenosine + H*_*2*_*O *→ *inosine + NH*_*3 *_and *inosine 5'-monophosphate + H*_*2*_*O *→ *inosine + orthophosphate*. (ids 74235 and 73822)

The events that precede 73822 are 73797 and 76590. However, these events are *not *part of the inosine formation pathway and the event that is listed in the pathway does not supply inputs to event 73822. Thus, the inosine formation pathway is not yet complete.

Overall, we found that 8 of the 55 pathways in Reactome were incomplete, and that there were 14 events in those 8 pathways that were the cause of the judgments of incompleteness. Since there are 742 events in these 55 pathways, these 14 events constitute 2% of all events.

### Consistency

Our tests identified a number of inconsistencies in Reactome. One example is the ornithine metabolism pathway.

The *ornithine metabolism *pathway (id 70693) has 9 events. Of these, event 70577 (*ATP + aspartate + citrulline *↔ *argininosuccinate + AMP + pyrophosphate*) is listed directly before 70560 (*carbamoyl phosphate + ornithine *→ *citrulline + orthophosphate*) in the pathway ordering. However, the preceding event of 70577 is 70560 according to the preceding event relationship specified in Reactome. This assertion is therefore inconsistent with the pathway ordering.

Eight of the 55 pathways had consistency problems and two of these are incomplete pathways. There were 23 reactions that were the cause of these inconsistencies and these 23 reactions comprise about 3% of all reactions.

### Gap-free pathways

There were very few gapped pathways in Reactome. Of the 55 concrete human pathways, only 4 were found to have gaps. There were 5 events in release 10 (0.6 % of all reactions) which could supply the necessary patches to seal the gaps.

Consider the *glycogen breakdown in liver cells *pathway (id 71598), which has 7 events. Event 71593 in the pathway requires{*(1,6)-alpha-glucosyl}poly{(1,4)-alpha-glucosyl}glycogenin-2 *as input, which is not provided by any of the previous events in the pathway. However, it can be supplied by event 71594, which is listed as its preceding event but is not considered part of the pathway.

### Directness (terseness and verbosity)

Our test for terseness revealed that only 3 of the 55 pathways were too terse and in each case, there was only one occurrence of terseness.

Again, consider the *glycogen breakdown in liver cells *pathway (id 71598). There are two events between events 71591 and 71593 according to the preceding event relationship.

Upon testing for verbosity, 18 of the 55 pathways in Reactome release 10 were found to be verbose.

Consider the *xanthine formation *pathway (id 74257), event 74249 and 75251 directly precedes event 74255 according to the preceding event relationship. However, the *xanthine formation *pathway lists four events 74248, 74249, 74250, 74251 grouped in the *guanine formation *pathway (id 74252) as occurring either in parallel or before event 74255.

We assert that verbosity is not that serious a problem, because excessive verbosity indicates that descriptions of pathways by domain experts can include extra steps for which evidence of the exact sequence of events is not explicitly specified or known. Although not optimal, this situation is not surprising for a biological process still under investigation.

### Well-formedness

On testing for well-formedness between consecutive events in pathways, we found that 21 of the 55 Reactome pathways were completely well-formed and 25 pathways presented 80% or more of their constituent events in well-formed sequences (80% well-formed). Table 3 shows the number of pathways at each level of well-formedness. Overall, 36 of the 55 pathways were at least 50% well-formed.

Consider the *Pyrimidine biosynthesis *pathway (id 73648). Event 73629 produces all the required inputs for event 75124 and hence supplies event 75124. However, event 73629 is not considered event 75124's preceding event. This violates well-formedness.

### Well-foundedness

There were no concrete reactions that violated this property.

**Figure 1:**
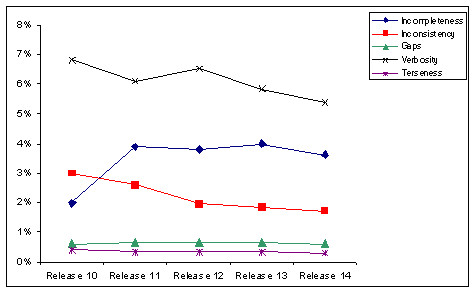
**Plot of the properties over Reactome releases**. The figure shows the change in the percentage of reactions that were responsible for the violation of each particular property from releases 10 to 14.

Reactome itself is constantly changing and evolving. This raises the possibility of studying how pathways evolve over time as curation progresses. Table 1 shows the comparison of release 10 through 14 in terms of the properties tested. Because pathways contain different numbers of reactions, it is more informative to compare the number of reactions for which a particular property was violated with the total number of reactions in that release rather than to directly compare the number of pathways with property violations. Table 2 shows the properties compared across releases based on the percentage of reactions in each release that were responsible for the violation of a particular property. Figure 1 shows a plot of the trends. We observe that inconsistencies are steadily declining along with the increase in the size of the knowledgebase. There is an increase in incompleteness as the number of pathways increases and new, possibly less characterised, pathways are added. Table 3 lists the numbers of well-formed pathways at various cut-offs in the successive release and we see that the percentage of well-formed pathways is steadily increasing over the releases.

## Discussion

We are developing computer-aided information integration and inference tools such as the Hypothesis Space Browser (HyBrow)[9,11]. In order for such tools to be useful, they should be able to use existing sources of structured information directly, rather than requiring users to create custom knowledgebases. The structured information stored in existing pathway knowledgebases is an obvious resource that can be used to serve such tools. The pathway resource list (PRL)[13] currently lists about 210 such pathway knowledgebases, indicating that a wealth of such information is becoming available.

We believe that evaluating pathway knowledgebases by proofreading and assessing their compatibility as well as a level of support for tools such as HyBrow is an important step toward the overall goal of using knowledgebases as effective resources for tools that perform information integration and computer-aided inference about biological processes.

The reliability of a knowledgebase for supporting information integration and computer-aided inference can be estimated by the kinds of tests outlined in the current work. A reliable pathway knowledgebase should be free of internal conflicts, represent as many steps as possible in each pathway, and provide the most complete set of pathway descriptions possible. Omissions, inconsistencies, errors in the order of steps in a pathway, missing steps, and extra steps all limit the performance of a knowledgebase. We believe that a reliable knowledgebase should minimally be complete, consistent, direct, gap-free, well-formed and well-founded. In this work, we provided precise mathematical definitions for each of these necessary properties and presented a logical framework for assessing a knowledgebase's reliability. We applied our tests to the latest releases of a deployed pathway knowledgebase and thereby demonstrated how these tests can be used to proofread and monitor the quality of a knowledgebase while it is being developed.

Reliability is, of course, not the whole story. To allow comparison of alternative representations of biological processes across organisms and databases, it is essential to have formal mechanisms to compare the richness and differences among representations of biological processes across existing knowledgebases. Even a completely reliable knowledgebase may lack the ability to support queries such as type-checking of stored relationships or be unable to capture certain kinds of biological relationships such as context dependent changes in the function of proteins.

In future work, we will evaluate a spectrum of knowledgebases for the purpose of comparing their expressive power. The situation we face when comparing different knowledgebases is complicated by the fact that the knowledgebase languages may not be in one-to-one correspondence. One approach is to consider only the subset of the language that many knowledgebases can express in a common interchange format, such as BioPAX. Alternately, we may choose to formulate a definition of expressiveness for comparing the expressiveness of knowledgebases given their (possibly implicit) ontologies and associated logics over their distinct languages. Our model theory formalism allows for rigorous comparisons of expressiveness. However, further work is required to examine which particular approach will yield the most meaningful comparisons.

## Conclusion

We used a model theoretic approach to check a deployed and maturing knowledgebase with respect to its suitability for machine-aided reasoning. We identified a desirable set of logical properties a pathway knowledgebase should satisfy to support machine-aided reasoning, including consistency, completeness and other desirable properties. Checking these properties during the design stage can predict and eliminate errors, as we demonstrated in this work.

We tested pathways from the available releases of Reactome for the satisfaction of our criteria. We implemented "proofreading" scripts to make our tests available for future use in the curation process. After reviewing our results, the Reactome team not only decided to make specific changes in response to errors we found, but also chose to incorporate our proofreading methods and scripts into their pre-release quality control process.

## Methods

In previous work, we developed a model-theoretic framework for representing and reasoning about biological systems[9,11]. A logic associated with a formal language can provide a set of rules for constructing and evaluating formulas, which are testable expressions using terms from the language. In model theory, a model is a formal representation of entities, relations, and the transformations among them[14]. For every expression constructed using the logic associated with the formal language, a model provides a testing environment with respect to the logic[11,15].

The Reactome data format implicitly generates a formal language. Elements from this formal language can be used to express formal models. If we can represent the information in the Reactome knowledgebase with a collection of formal models representing reactions, pathways and collections of pathways, we can then perform model checking to proofread it. Specifically, we can identify desirable logical properties for models representing pathways and develop tests to perform model-checking and query evaluation to verify these properties.

This approach allows us to cast knowledgebase verification in a broader context while building a strong connection to our previous work on computer-aided hypothesis composition and evaluation done by HyBrow[11]. On the practical side, this approach allowed us to verify a potential data source's compatibility with our evaluation environment. Building a logic for verifying representations of pathways in knowledgebases will also allow us to extend our HyBrow framework for phrasing and testing hypotheses at higher levels of abstraction, a topic we intend to pursue in future work.

### A formal language for Reactome

Reactome contains representations of both concrete and generic reactions, and this investigation focuses on the former. A concrete reaction is a reaction in which all inputs, outputs, and catalysts are concrete physical entities (entities that represent a single instance of a gene, protein or chemical), and in which the conversion of inputs to outputs occurs in a single step.

In addition to inputs and outputs, a reaction can include information on the organism, sub-cellular location and the experimental evidence for the reaction in the form of one or more literature citations, as well as catalytic activity and regulatory information. Reactome's definition of reaction encompasses classical biochemical reactions (for example, the phosphorylation of glucose to glucose-6-phosphate), as well as events such as binding, dissociation, complex formation, translocation and conformational changes.

The defining attributes of a reaction are its input, output, and catalyst activity. Reactions which have identical substrates and products but different catalysts are represented as distinct reactions. Similarly, chemically identical entities in different cellular compartments are represented as distinct entities. For example, extracellular and cytosolic glucose are stored as separate entities. Entities in different biochemical modification states are also represented as separate entities. The p53 protein, for example, is represented by three distinct entities: native p53, p53 phosphorylated at Ser15 and p53 phosphorylated at Ser20. Such multiple state representations are derived from a single base entity – what Reactome calls the Reference Entity – which contains information common to all the states.

Reactome events can also contain generic physical entities such as 'any tRNA'[2]. Reactions are grouped by Reactome into pathways[2] that take into account the reactions' temporal relationships and interdependencies. Pathways in Reactome are groupings of functionally related reactions, and can contain sequential reactions, parallel reactions or reactions ordered in a cycle[2]. Pathways can also nest. That is, pathways can have other pathways as their components and can be sequential or parallel[2]. A concrete pathway is a multi-step concrete event whose components are concrete reactions, concrete pathways, or both. If a concrete pathway contains sequential reactions, they are displayed in the order they occur. Reactome also stores a specific 'preceding event' relationship that defines the exact order of two reactions.

In this paper we restrict the use of the term *event *to concrete reactions and use the term *pathway *for a concrete pathway. In order to specify a formal language for a logic, we need a set of functions, a set of relations and a set of constants.

For this work, a formal language *L *is a triple *L *= <*F*, *R*, *C>* where:

• *F *is a set of function symbols, each with positive integer arity,

• *R *is a set of relation symbols, each with non-negative integer arity, and

• *C *is a set of constant symbols.

The concepts defined by Reactome can satisfy the requirements of a formal language if we make the following translations of Reactome concepts.

• Reactome's representations of physical entities provide the constant symbols of the formal language.

• Reactions that transform one version of a physical entity into another version, form or dissociate complexes, or transport entities into different contexts form the functions of the formal language.

• Reactions not otherwise specified as functions form relations in the formal language. In particular, temporal relationships stored by Reactome are expressed using the 'precedes' relation.

### Event relationships in Reactome

The base event-level relationship in Reactome is temporal precedence, which is directly stored by Reactome for each reaction. We supplement the 'precedes' relation by defining a set of additional event-level relations that allow us to define testable properties at the pathway level.

• We consider an event e_n _to be *directly supplied *by event e_n-1 _if the inputs of e_n _are included among the outputs of e_n-1_. We consider it partially supplied by event e_n-1 _if at least one of the inputs of e_n _is present among the outputs of e_n-1_. According to Reactome, partially supplying the following event is a requirement for an event to precede another event.

• We consider an event e_n _to be *indirectly supplied *by events e_1 _... e_n-1 _if all the inputs of e_n _are contained in the union of the outputs of the other events

Supply is the union of direct, partial and indirect supply.

• We consider an event e_n _to be *enabled *by event e_i _(or events e_1_...e_n-1_) if all of the catalysts of e_n _are found among the outputs of e_i _(or events e_1_...e_n-1_).

### Models from a formal language

The language we derived from the Reactome data format and the event relationships defined above allow us to specify pathway-level models[14] in the formalism of model theory. Let L be a language. A *model *(or L-structure) is a tuple M = <*M*, *F*^*M*^, *R*^*M*^, *C*^*M*^*>*, where *M *is a set called the universe containing the objects from L. The universe is closed under function operation. *F*^*M *^is a set of functions, *R*^*M *^is a set of relations, and *C*^*M *^is a set of constants in *M*.

We focus our attention on finite models[11]. These can represent anything from an event, to a pathway, to a set of pathways such as "Insulin receptor mediated signalling," by instantiating a hierarchy of constants, relations, and functions.

For Reactome pathway-models, the constants are individual Reactome events. We define the 'enable' and 'supply' relationships as above, and also use the 'precedes' relation as defined by Reactome. The universe for such models is the set of all Reactome events, together with the "initial conditions" which provide all of the entities assumed to be axiomatically present.

### A logic for checking pathway-models

Model-checking requires two inputs: a model and a formula. Given a language L, a model M and an L-formula φ, solving the model checking problem answers the question of whether φ is true in M. Given a model M and a formula φ (x) with free variables x, solving the query evaluation problem computes the relation defined by φ on M (the setφ^*M*^: = {*a *∈ *M*: *M*| = φ(*a*)}). Finding all pathway-models that exhibit a specific property, such as being complete, is a query-evaluation problem. (A query evaluation can be converted to a set of model-checking problems).

To construct a logical framework for checking pathway-models we formalize an analogy between truth and presence of an entity. We then apply a logic capable of deciding truth values in order to verify which compounds could be present at which times. In traditional logic, we evaluate 'truth' using deduction rules.

For instance, the classical deduction rule, "modus ponens," is given as:

ϕ,ϕ⇒ψψ,
 MathType@MTEF@5@5@+=feaafiart1ev1aaatCvAUfKttLearuWrP9MDH5MBPbIqV92AaeXatLxBI9gBaebbnrfifHhDYfgasaacH8akY=wiFfYdH8Gipec8Eeeu0xXdbba9frFj0=OqFfea0dXdd9vqai=hGuQ8kuc9pgc9s8qqaq=dirpe0xb9q8qiLsFr0=vr0=vr0dc8meaabaqaciaacaGaaeqabaqabeGadaaakeaadaWcaaqaaiabew9aQjabcYcaSiabew9aQjabgkDiElabeI8a5bqaaiabeI8a5baacqGGSaalaaa@380D@

which says that given the truth of formulas φ and φ =>ψ, we can deduce the truth of ψ.

We have defined a set of rules analogous to the deduction rules of traditional logic. This new set of rules, which we call verification rules to avoid confusion with the traditional deduction rules, takes the place of the standard deduction rules in evaluating Reactome models. We define such rules for supply and enabling in order to evaluate whether or not a given entity can be present given the reactions that have happened so far.

For example, we have defined the following set of verification rules for verifying supply in Reactome.

Using the syntax I→RCO
 MathType@MTEF@5@5@+=feaafiart1ev1aaatCvAUfKttLearuWrP9MDH5MBPbIqV92AaeXatLxBI9gBaebbnrfifHhDYfgasaacH8akY=wiFfYdH8Gipec8Eeeu0xXdbba9frFj0=OqFfea0dXdd9vqai=hGuQ8kuc9pgc9s8qqaq=dirpe0xb9q8qiLsFr0=vr0=vr0dc8meaabaqaciaacaGaaeqabaqabeGadaaakeaacqWGjbqscqGHsgIRdaqhaaWcbaGaemOuaifabaGaem4qameaaOGaem4ta8eaaa@334E@ to describe an event consisting of a reaction R on a set of inputs I, leading to the generation of a set of outputs O with the aid of catalysts C:

1. I→RCO,present(I),present(C)present(O)
 MathType@MTEF@5@5@+=feaafiart1ev1aaatCvAUfKttLearuWrP9MDH5MBPbIqV92AaeXatLxBI9gBaebbnrfifHhDYfgasaacH8akY=wiFfYdH8Gipec8Eeeu0xXdbba9frFj0=OqFfea0dXdd9vqai=hGuQ8kuc9pgc9s8qqaq=dirpe0xb9q8qiLsFr0=vr0=vr0dc8meaabaqaciaacaGaaeqabaqabeGadaaakeaadaWcaaqaaiabdMeajjabgkziUoaaDaaaleaacqWGsbGuaeaacqWGdbWqaaGccqWGpbWtcqGGSaalcqWGWbaCcqWGYbGCcqWGLbqzcqWGZbWCcqWGLbqzcqWGUbGBcqWG0baDdaqadaqaaiabdMeajbGaayjkaiaawMcaaiabcYcaSiabdchaWjabdkhaYjabdwgaLjabdohaZjabdwgaLjabd6gaUjabdsha0naabmaabaGaem4qameacaGLOaGaayzkaaaabaGaemiCaaNaemOCaiNaemyzauMaem4CamNaemyzauMaemOBa4MaemiDaq3aaeWaaeaacqWGpbWtaiaawIcacaGLPaaaaaaaaa@5A4D@;

2. I→R1C1O,O→R2C2O2,precedes(R1,R2)I→Rnew(C1∪C2)O2
 MathType@MTEF@5@5@+=feaafiart1ev1aaatCvAUfKttLearuWrP9MDH5MBPbIqV92AaeXatLxBI9gBaebbnrfifHhDYfgasaacH8akY=wiFfYdH8Gipec8Eeeu0xXdbba9frFj0=OqFfea0dXdd9vqai=hGuQ8kuc9pgc9s8qqaq=dirpe0xb9q8qiLsFr0=vr0=vr0dc8meaabaqaciaacaGaaeqabaqabeGadaaakeaadaWcaaqaaiabdMeajjabgkziUoaaDaaaleaacqWGsbGucqaIXaqmaeaacqWGdbWqcqaIXaqmaaGccqWGpbWtcqGGSaalcqWGpbWtcqGHsgIRdaqhaaWcbaGaemOuaiLaeGOmaidabaGaem4qamKaeGOmaidaaOGaem4ta8KaeGOmaiJaeiilaWIaemiCaaNaemOCaiNaemyzauMaem4yamMaemyzauMaemizaqMaemyzauMaem4Cam3aaeWaaeaacqWGsbGucqaIXaqmcqGGSaalcqWGsbGucqaIYaGmaiaawIcacaGLPaaaaeaacqWGjbqscqGHsgIRdaqhaaWcbaGaemOuai1aaSbaaWqaaiabd6gaUjabdwgaLjabdEha3bqabaaaleaadaqadaqaaiabdoeadjabigdaXiabgQIiilabdoeadjabikdaYaGaayjkaiaawMcaaaaakiabd+eapjabikdaYaaaaaa@641A@;

Thus, we "verify" that the outputs of the reaction are present only if all of a reaction's required inputs and catalysts are present, established by preceding reactions or assumed (axiomatically) as initial conditions.

We define a verification to be a finite sequence φ_0_, φ_1_, ..., φ_n _such that φ_i _is obtained from an axiom, or is obtained from φ_0_, ...,φ_i-1 _through the use of a verification rule. Establishing verification rules as described above allows us to check pathway-models to determine whether or not they satisfy the definitions of the pathway-level properties described in the next section. For example, it is desirable that all of the agents required in a certain pathway be supplied either as initial conditions to that pathway or by events upstream.

## Authors' contributions

SR conceived the theory and developed the tests, NS programmed the tests and compiled the results, and all authors were involved in the writing and editing of this manuscript.

## Figures and Tables

**Table 1:** Comparison of Reactome releases. The table shows the number of pathways that satisfy each property tested.

Property	Release 10	Release 11	Release 12	Release 13	Release 14
Total pathways	55	65	64	61	64
Complete pathways	47	51	51	47	51
Consistent pathways	47	54	56	54	57
Gap-free pathways	51	60	59	56	59
Non-verbose pathways	37	44	42	41	44
Non-terse pathways	52	62	61	58	61
90 % well-formed	21	27	29	27	30
Unfounded	0	0	0	0	0

**Table 2:** Comparison of properties at the reaction level over Reactome releases. The table shows the percentage of reactions in a particular release which were responsible for the violation of a particular property.

Property	Release 10	Release 11	Release 12	Release 13	Release 14
Incompleteness	2.00 %	3.90 %	3.80 %	4.00 %	3.60 %
Inconsistency	3.00 %	2.60 %	1.95 %	1.84 %	1.70 %
Gaps	0.60 %	0.64 %	0.65 %	0.65 %	0.60 %
Verbosity	6.80 %	6.10 %	6.52 %	5.85 %	5.40 %
Terseness	0.40 %	0.32 %	0.32 %	0.32 %	0.30 %
90% Well formed	40.00 %	42.00 %	45.00 %	44.00 %	47.00 %
Unfounded	0.00 %	0.00 %	0.00 %	0.00 %	0.00 %

**Table 3:** Well-formed pathways. Numbers of Well-formed pathways at different levels of well-formedness for the latest releases. The numbers in the brackets are the percentages of total pathways. Release 10, 11, 12, 13 and 14 contain 55, 65, 64, 61 and 64 pathways respectively.

Percentage well formed	Release 10	Release 11	Release 12	Release 13	Release 14
100	21 [38%]	26 [40%]	29 [45%]	27 [44%]	30 [47%]
90	22 [40%]	27 [42%]	29 [45%]	27 [44%]	30 [47%]
80	25 [45%]	30 [46%]	33 [52%]	31 [51%]	34 [53%]
70	27 [49%]	33 [50%]	36 [56%]	34 [56%]	37 [58%]
60	33 [60%]	40 [62%]	43 [67%]	41 [67%]	44 [69%]
50	36 [65%]	43 [66%]	48 [75%]	46 [75%]	49 [77%]
40	37 [67%]	44 [68%]	50 [78%]	49 [80%]	52 [81%]
